# Supplementary material for: Functional diversification of horizontally acquired glycoside hydrolase family 45 (GH45) proteins in Phytophaga beetles
Source: BMC Evol Biol. 2019 May 10;19:100. doi: 10.1186/s12862-019-1429-9 (PMC6509783; doi:10.1186/s12862-019-1429-9)
Supplement: Supplementary file 1 — Table S1 to S4; Figs. S1 to S10 (legends included). (DOCX 22341 kb) [file 12862_2019_1429_MOESM1_ESM.docx]

**Additional file 1**

**Functional analyses of the horizontally acquired Phytophaga glycoside hydrolase family 45 (GH45) proteins reveal distinct functional characteristics**

André Busch^1^, Etienne G.J. Danchin^2^ and Yannick Pauchet^1*^

**Contains:**

- Supplementary tables
- Supplementary figures

**Table S1.** Details on the beetle-derived GH45 proteins that were expressed in *Sf*9 cells.

| **Acronym** | **Species** | **Family** | **Subfamily** | **Accession** | **Reference** |
| --- | --- | --- | --- | --- | --- |
| CTR1 | *Chrysomela tremula* | Chrysomelidae | Chrysomelinae | ADU33285.1 | (Pauchet, et al. 2010) |
| CTR2 | *Chrysomela tremula* | Chrysomelidae | Chrysomelinae | ADU33286.1 | (Pauchet, et al. 2010) |
| CTR3 | *Chrysomela tremula* | Chrysomelidae | Chrysomelinae | MH892457 | This study |
| LDE1 | *Leptinotarsa decemlineata* | Chrysomelidae | Chrysomelinae | ADU33345.1 | (Pauchet, et al. 2010) |
| LDE2 | *Leptinotarsa decemlineata* | Chrysomelidae | Chrysomelinae | ADU33346.1 | (Pauchet, et al. 2010) |
| LDE3 | *Leptinotarsa decemlineata* | Chrysomelidae | Chrysomelinae | ADU33347.1 | (Pauchet, et al. 2010) |
| LDE4 | *Leptinotarsa decemlineata* | Chrysomelidae | Chrysomelinae | ADU33348.1 | (Pauchet, et al. 2010) |
| LDE5 | *Leptinotarsa decemlineata* | Chrysomelidae | Chrysomelinae | ADU33349.1 | (Pauchet, et al. 2010) |
| LDE6 | *Leptinotarsa decemlineata* | Chrysomelidae | Chrysomelinae | ADU33350.1 | (Pauchet, et al. 2010) |
| LDE7 | *Leptinotarsa decemlineata* | Chrysomelidae | Chrysomelinae | ADU33351.1 | (Pauchet, et al. 2010) |
| LDE8 | *Leptinotarsa decemlineata* | Chrysomelidae | Chrysomelinae | MH892458 | This study |
| LDE9 | *Leptinotarsa decemlineata* | Chrysomelidae | Chrysomelinae | MH892459 | This study |
| LDE10 | *Leptinotarsa decemlineata* | Chrysomelidae | Chrysomelinae | MH892460 | This study |
| LDE11 | *Leptinotarsa decemlineata* | Chrysomelidae | Chrysomelinae | MH892461 | This study |
| PCO1 | *Phaedon cochleariae* | Chrysomelidae | Chrysomelinae | HE962202.1 | (Kirsch, et al. 2012) |
| PCO3 | *Phaedon cochleariae* | Chrysomelidae | Chrysomelinae | HE962203.1 | (Kirsch, et al. 2012) |
| PCO4 | *Phaedon cochleariae* | Chrysomelidae | Chrysomelinae | HE962204.1 | (Kirsch, et al. 2012) |
| PCO5 | *Phaedon cochleariae* | Chrysomelidae | Chrysomelinae | HE962205.1 | (Kirsch, et al. 2012) |
| PCO6 | *Phaedon cochleariae* | Chrysomelidae | Chrysomelinae | HE962206.1 | (Kirsch, et al. 2012) |
| PCO7 | *Phaedon cochleariae* | Chrysomelidae | Chrysomelinae | HE962207.1 | (Kirsch, et al. 2012) |
| PCO8 | *Phaedon cochleariae* | Chrysomelidae | Chrysomelinae | HE962208.1 | (Kirsch, et al. 2012) |
| DVI1 | *Diabrotica vir. virgifera* | Chrysomelidae | Galerucinae | AFI56547.1 | (Valencia, et al. 2013) |
| DVI2 | *Diabrotica vir. virgifera* | Chrysomelidae | Galerucinae | MH892463 | (Eyun, et al. 2014) |
| DVI3 | *Diabrotica vir. virgifera* | Chrysomelidae | Galerucinae | MH892464 | (Eyun, et al. 2014) |
| DVI4 | *Diabrotica vir. virgifera* | Chrysomelidae | Galerucinae | MH892465 | (Eyun, et al. 2014) |
| DVI5 | *Diabrotica vir. virgifera* | Chrysomelidae | Galerucinae | MH892466 | (Eyun, et al. 2014) |
| DVI6 | *Diabrotica vir. virgifera* | Chrysomelidae | Galerucinae | MH892467 | (Eyun, et al. 2014) |
| DVI7 | *Diabrotica vir. virgifera* | Chrysomelidae | Galerucinae | MH892468 | (Eyun, et al. 2014) |
| DVI8 | *Diabrotica vir. virgifera* | Chrysomelidae | Galerucinae | MH892469 | (Eyun, et al. 2014) |
| DVI9 | *Diabrotica vir. virgifera* | Chrysomelidae | Galerucinae | MH892470 | (Eyun, et al. 2014) |
| DVI10 | *Diabrotica vir. virgifera* | Chrysomelidae | Galerucinae | MH892471 | (Eyun, et al. 2014) |
| DVI11 | *Diabrotica vir. virgifera* | Chrysomelidae | Galerucinae | MH892472 | (Eyun, et al. 2014) |
| SOR1 | *Sitophilus oryzae* | Curculionidae | Dryophtorinae | ADU33246.1 | (Pauchet, et al. 2010) |
| SOR2 | *Sitophilus oryzae* | Curculionidae | Dryophtorinae | ADU33247.1 | (Pauchet, et al. 2010) |
| SOR3 | *Sitophilus oryzae* | Curculionidae | Dryophtorinae | ADU33248.1 | (Pauchet, et al. 2010) |
| SOR4 | *Sitophilus oryzae* | Curculionidae | Dryophtorinae | ADU33249.1 | (Pauchet, et al. 2010) |
| SOR5 | *Sitophilus oryzae* | Curculionidae | Dryophtorinae | ADU33250.1 | (Pauchet, et al. 2010) |

**References**

Eyun SI, Wang H, Pauchet Y, ffrench-Constant RH, Benson AK, Valencia-Jimenez A, Moriyama EN, Siegfried BD. 2014. Molecular evolution of glycoside hydrolase genes in the western corn rootworm (*Diabrotica virgifera virgifera*). PLoS ONE 9:e94052.

Kirsch R, Wielsch N, Vogel H, Svatos A, Heckel DG, Pauchet Y. 2012. Combining proteomics and transcriptome sequencing to identify active plant-cell-wall-degrading enzymes in a leaf beetle. BMC Genomics 13:587.

Pauchet Y, Wilkinson P, Chauhan R, Ffrench-Constant RH. 2010. Diversity of beetle genes encoding novel plant cell wall degrading enzymes. PLoS ONE 5:e15635.

Valencia A, Alves AP, Siegfried BD. 2013. Molecular cloning and functional characterization of an endogenous endoglucanase belonging to GHF45 from the western corn rootworm, *Diabrotica virgifera virgifera*. Gene 513:260-267.

**Table S2.** Details of the sequences used for the large phylogenetic analyses.

**Table S3.** Details on the genome/transcriptome datasets which were used to curate GH45 sequences derived from Phytophaga beetles.

| **Species** | **Acronym** | **Superfamily** | **family** | **subfamily** | **Data type^*^** | **accession** |
| --- | --- | --- | --- | --- | --- | --- |
| *Cylas brunneus* | CBR | Curculionoidea | Brentidae | Brentinae | SRA | SRX1710181 |
| *Cylas formicarius* | CFO | Curculionoidea | Brentidae | Brentinae | SRA | SRX1508049 |
| *Cylas puncticollis* | CPU | Curculionoidea | Brentidae | Brentinae | SRA | SRX732288 |
|  |  |  |  |  |  |  |
| *Anthonomus grandis* | AGR | Curculionoidea | Curculionidae | Curculioninae | SRA | SRX2888367 |
| *Listronotus oregonensis* | LOR | Curculionoidea | Curculionidae | Cyclominae | SRA | SRX1674531; SRX1674530; SRX1674529 |
| *Cyrtotrachelus buqueti* | CBU | Curculionoidea | Curculionidae | Dryophthorinae | SRA | SRX3262946 |
| *Rhynchophorus ferrugineus* | RFE | Curculionoidea | Curculionidae | Dryophthorinae | TSA | GDKA00000000.1 |
| *Sitophilus oryzae* | SOR | Curculionoidea | Curculionidae | Dryophthorinae | SRA | SRX017240 |
| *Sphenophorus levis* | SLE | Curculionoidea | Curculionidae | Dryophthorinae | Sanger ESTs | JZ135722.1-JZ139168.1 |
| *Diaprepes abbreviatus* | DAB | Curculionoidea | Curculionidae | Entiminae | Sanger ESTs | CN472512.1-CN488395.1; DN199437.1-DN201109.1 |
| *Pachyrhynchus infernalis* | PIN | Curculionoidea | Curculionidae | Entiminae | SRA | DRX089461; DRX089463; DRX089465 |
| *Hylobius abietis* | HAB | Curculionoidea | Curculionidae | Molytinae | SRA | SRX3423630 |
| *Larinus minutus* | LMI | Curculionoidea | Curculionidae | Molytinae | TSA | GDLA00000000.1 |
| *Pissodes strobi* | PST | Curculionoidea | Curculionidae | Molytinae | Sanger ESTs | GT285068.1-GT296156.1 |
| *Euwallacea fornicatus* | EFO | Curculionoidea | Curculionidae | Scolytinae | SRA | SRX698946 |
| *Hypothenemus hampei* | HHA | Curculionoidea | Curculionidae | Scolytinae | Genome | LBGY00000000.1 |
| *Ips pini* | IPI | Curculionoidea | Curculionidae | Scolytinae | Sanger ESTs | CB407474.1-CB409136.1 |
| *Ips typographus* | ITY | Curculionoidea | Curculionidae | Scolytinae | TSA | GACR00000000.1 |
| *Tomicus yunnanensis* | TYU | Curculionoidea | Curculionidae | Scolytinae | SRA | SRX2518383 |
| *Dendroctonus ponderosae* | DPO | Curculionoidea | Curculionidae | Scolytinae | Genome | APGK00000000.1 |
|  |  |  |  |  |  |  |
| *Anoplophora glabripennis* | AGL | Chrysomeloidea | Cerambycidae | Lamiinae | Genome | AQHT00000000.2 |
| *Apriona japonica* | AJA | Chrysomeloidea | Cerambycidae | Lamiinae | SRA | ERX387572-ERX387579 |
| *Monochamus alternatus* | MAL | Chrysomeloidea | Cerambycidae | Lamiinae | SRA | SRX1302202; SRX1605798; SRX1605842; SRX1606425; SRX1606426 |
| *Anoplophora chinensis* | ACH | Chrysomeloidea | Cerambycidae | Lamiinae | NR | AFN89565.1 |
| *Apriona germari* | AGE | Chrysomeloidea | Cerambycidae | Lamiinae | NR | AAU44973.1, AAR22385.1 |
| *Batocera horsfieldi* | BHO | Chrysomeloidea | Cerambycidae | Lamiinae | NR | AKH90729.1 |
| *Mesosa myops* | MMY | Chrysomeloidea | Cerambycidae | Lamiinae | NR | AMA76413.1 |
| *Oncideres albomarginata chamela* | OAL | Chrysomeloidea | Cerambycidae | Lamiinae | NR | ADI24132.1 |
| *Psacothea hilaris* | PHI | Chrysomeloidea | Cerambycidae | Lamiinae | NR | ALE71518.1 |
|  |  |  |  |  |  |  |
| *Cassida rubiginosa* | CRU | Chrysomeloidea | Chrysomelidae | Cassidinae | SRA | SRX3287590; SRX3287591; SRX3287593 |
| *Octodonta nipae* | ONI | Chrysomeloidea | Chrysomelidae | Cassidinae | SRA | SRX396790 |
| *Chrysomela tremula* | CTR | Chrysomeloidea | Chrysomelidae | Chrysomelinae | SRA | SRX017241 |
| *Chrysomela populi* | CPO | Chrysomeloidea | Chrysomelidae | Chrysomelinae | SRA | SRX390590; SRX390602; SRX390603 |
| *Colaphellus bowringi* | CBO | Chrysomeloidea | Chrysomelidae | Chrysomelinae | SRA | SRX317064 |
| *Gastrophysa viridula* | GVI | Chrysomeloidea | Chrysomelidae | Chrysomelinae | SRA | SRX017237 |
| *Leptinotarsa decemlineata* | LDE | Chrysomeloidea | Chrysomelidae | Chrysomelinae | SRA | SRX017239 |
| *Oreina cacaliae* | OCA | Chrysomeloidea | Chrysomelidae | Chrysomelinae | TSA | GDPL00000000.1 |
| *Phaedon cochleariae* | PCO | Chrysomeloidea | Chrysomelidae | Chrysomelinae | in-house transcriptome |  |
| *Clitea metallica* | CME | Chrysomeloidea | Chrysomelidae | Galerucinae | SRA | SRX3921909 |
| *Diabrotica virgifera virgifera* | DVI | Chrysomeloidea | Chrysomelidae | Galerucinae | TSA | GBSB00000000.1 |
| *Phyllotreta armoraciae* | PAR | Chrysomeloidea | Chrysomelidae | Galerucinae | in-house transcriptome |  |
| *Podagricomela weisei* | PWE | Chrysomeloidea | Chrysomelidae | Galerucinae | SRA | SRX3921907 |
| *Psylliodes chrysocephala* | PCH | Chrysomeloidea | Chrysomelidae | Galerucinae | in-house transcriptome |  |

^*^SRA: Short read archive at NCBI; TSA: transcriptome shotgun assembly at NCBI; NR: non redundant protein database at NCBI.

**Table S4.** List of primers used in this study.

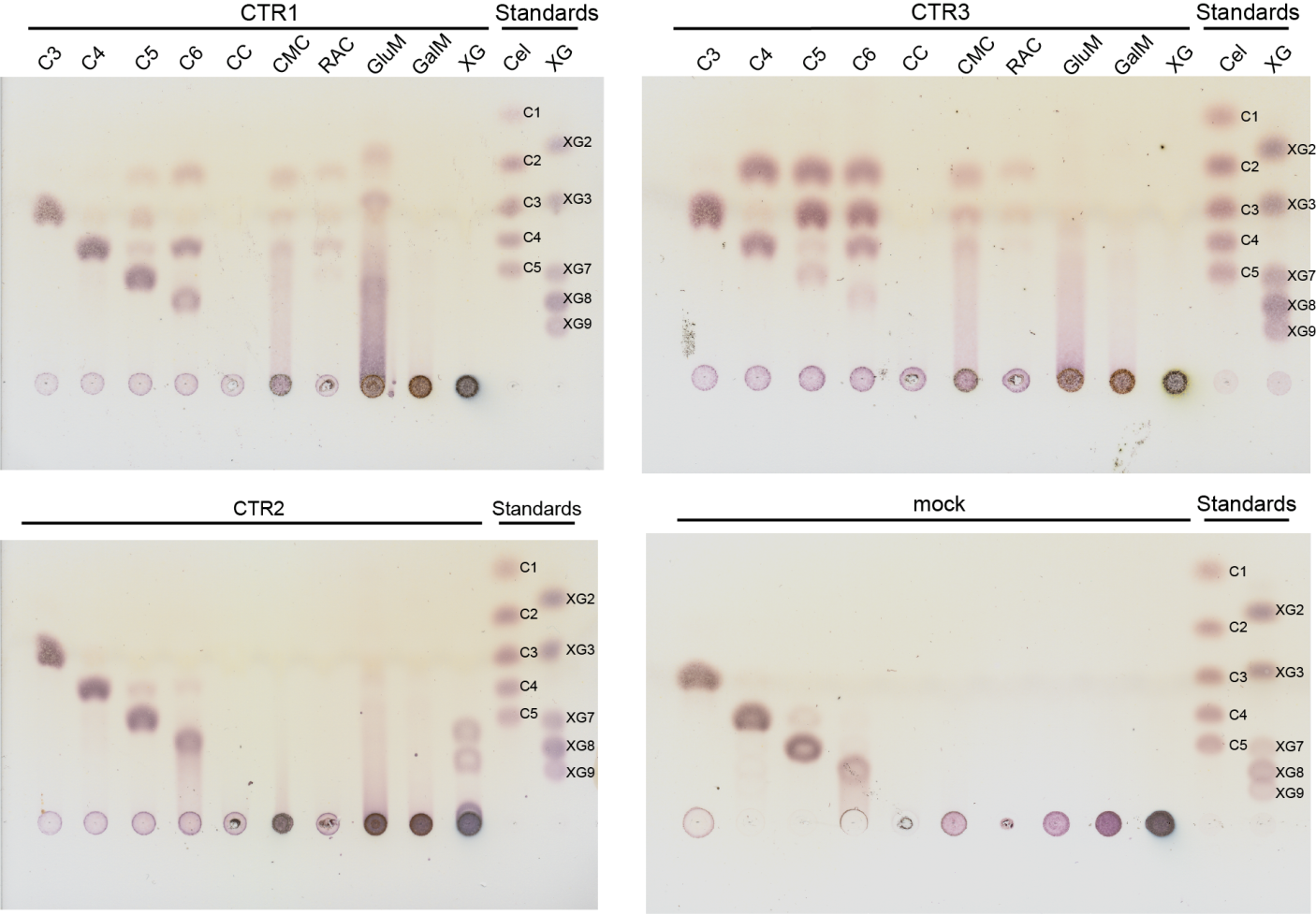
**Fig. S1 Thin-layer chromatography of *C. tremula* GH45s assayed against several plant cell wall polysaccharides.** Recombinant GH45s were incubated for 16 h at 40 °C with various plant polysaccharides. Their breakdown products were analyzed on TLC and visualized using 0.2 % orcinol in methane/sulphoric acid (9:1) under continuous heating. Each TLC represents an individually tested GH45 (Ctr1 to Ctr3). All GH45s were assayed against the same set of substrates, namely, cellotriose to cellohexaose (C3-C6); crystalline cellulose = avicel (CC); carboxymethyl cellulose (CMC); regenerated amorphous cellulose (RAC); glucomannan (GluM); galactomannan (GalM); xyloglucan (XG); standards: C1 = glucose, C2 – C5 = cellobiose to pentaose; XG2- XG9 = xyloglucan-oligomers.


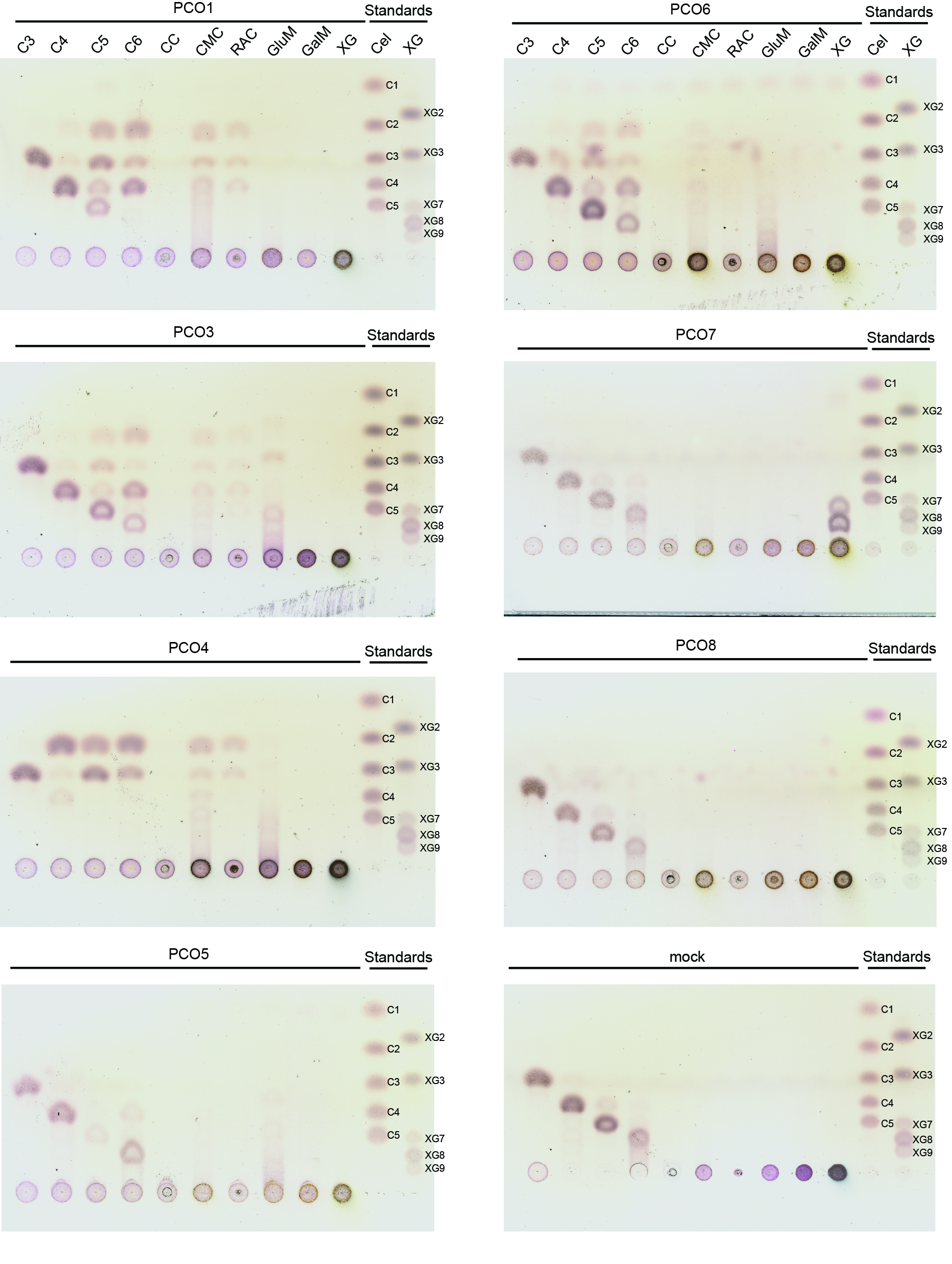


**Fig. S2 Thin layer chromatography of P. cochleariae GH45s assayed against several plant cell wall polysaccharides.** Recombinant GH45s were incubated for 16 h at 40 °C with various plantpolysaccharides. Their breakdown products were analyzed on TLC and visualized using 0.2 % orcinol in methane/sulphoric acid (9:1) under continuous heating. Each TLC represents an individually tested GH45 (Pco1 to Pco8). All GH45s were assayed against the same set of substrates which included cellotriose to cellohexaose (C3-C6); crystalline cellulose = avicel (CC); carboxymethyl cellulose (CMC); regenerated amorphous cellulose (RAC); glucomannan (GluM); galactomannan (GalM); xyloglucan (XG); standards: C1 = glucose, C2 – C5 = cellobiose – pentaose; XG2- XG9 = xyloglucan- oligomers.Fig. S1 Thin-layer chromatography of *C. tremula* GH45s assayed against several plant cell wall polysaccharides.


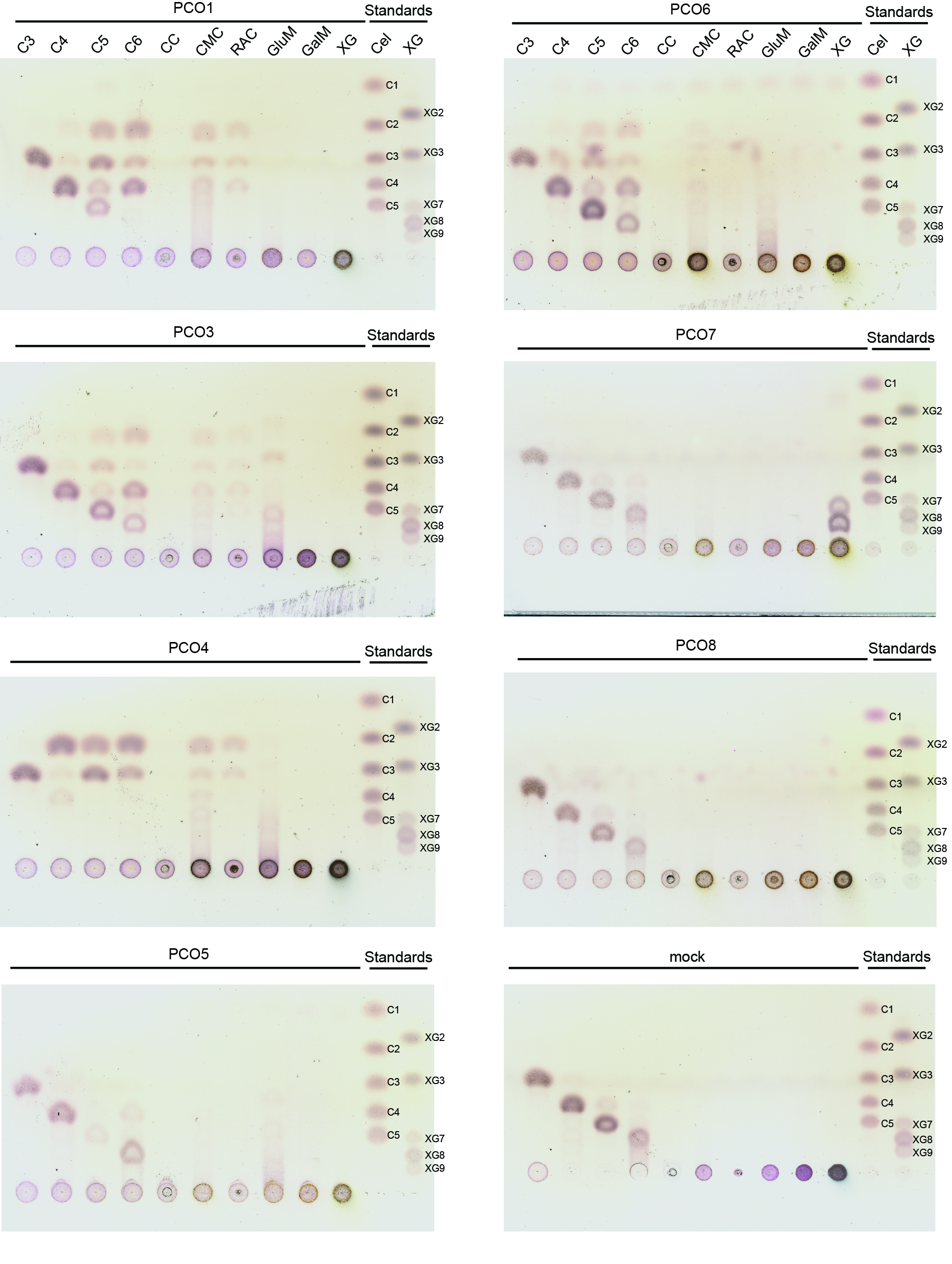


**Fig. S2 continued**


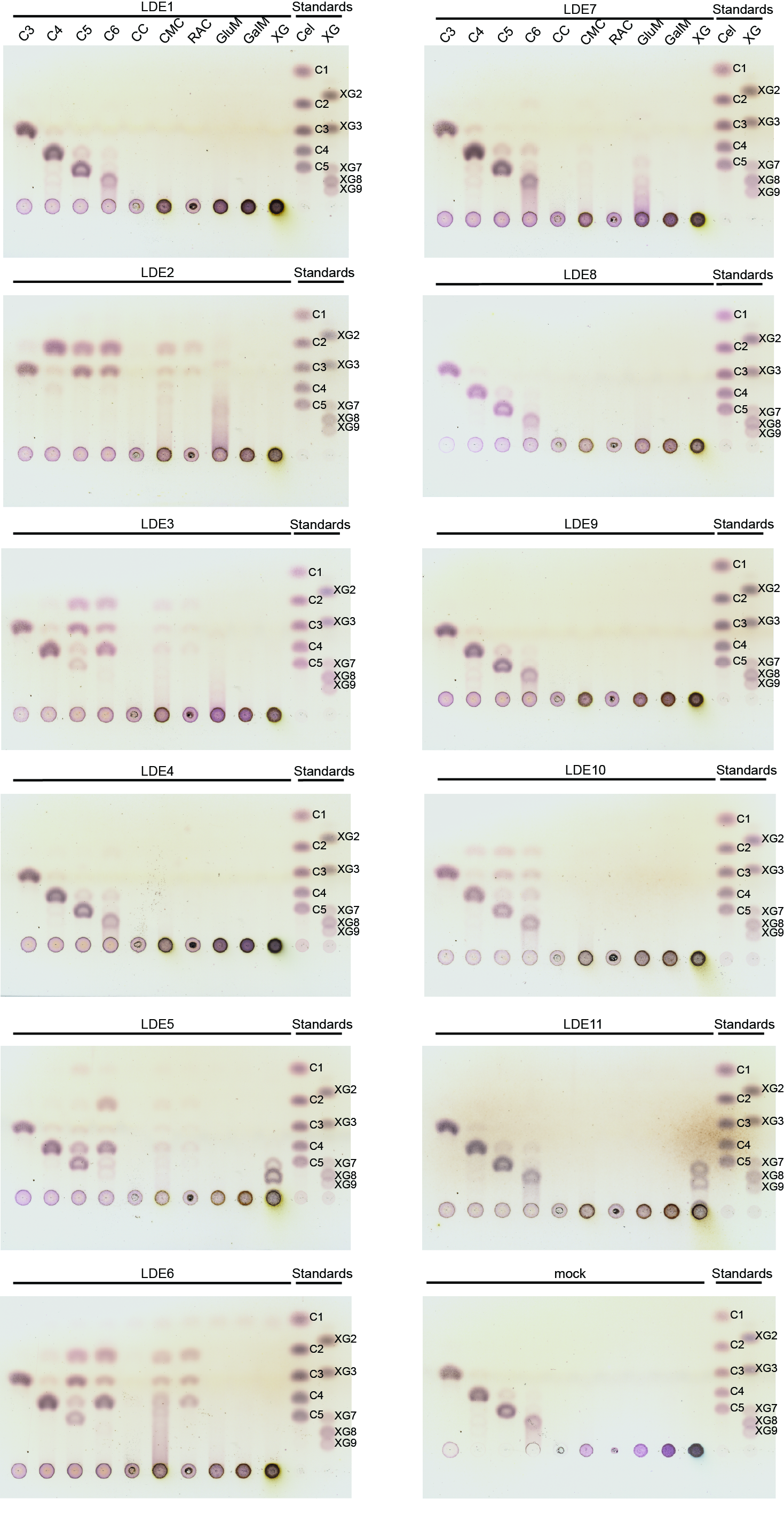


**Fig. S3 Thin layer chromatography of *L. decemlineata* GH45s assayed against several plant cell wall polysaccharides.** Recombinant GH45s were incubated for 16 h at 40 °C with various plant polysaccharides. Their breakdown products were analyzed on TLC and visualized using 0.2 % orcinol in methane/sulphoric acid (9:1) under continuous heating. Each TLC represents an individually tested GH45 (Lde1 to Lde11). All GH45s were assayed against the same set of substrates which included: cellotriose to cellohexaose (C3-C6); crystalline cellulose = avicel (CC); carboxymethyl cellulose (CMC); regenerated amorphous cellulose (RAC); glucomannan (GluM); galactomannan (GalM); xyloglucan (XG); standards: C1 = slucose, C2 – C5 = cellobiose – pentaose; XG2- XG9 = xyloglucan- oligomers.


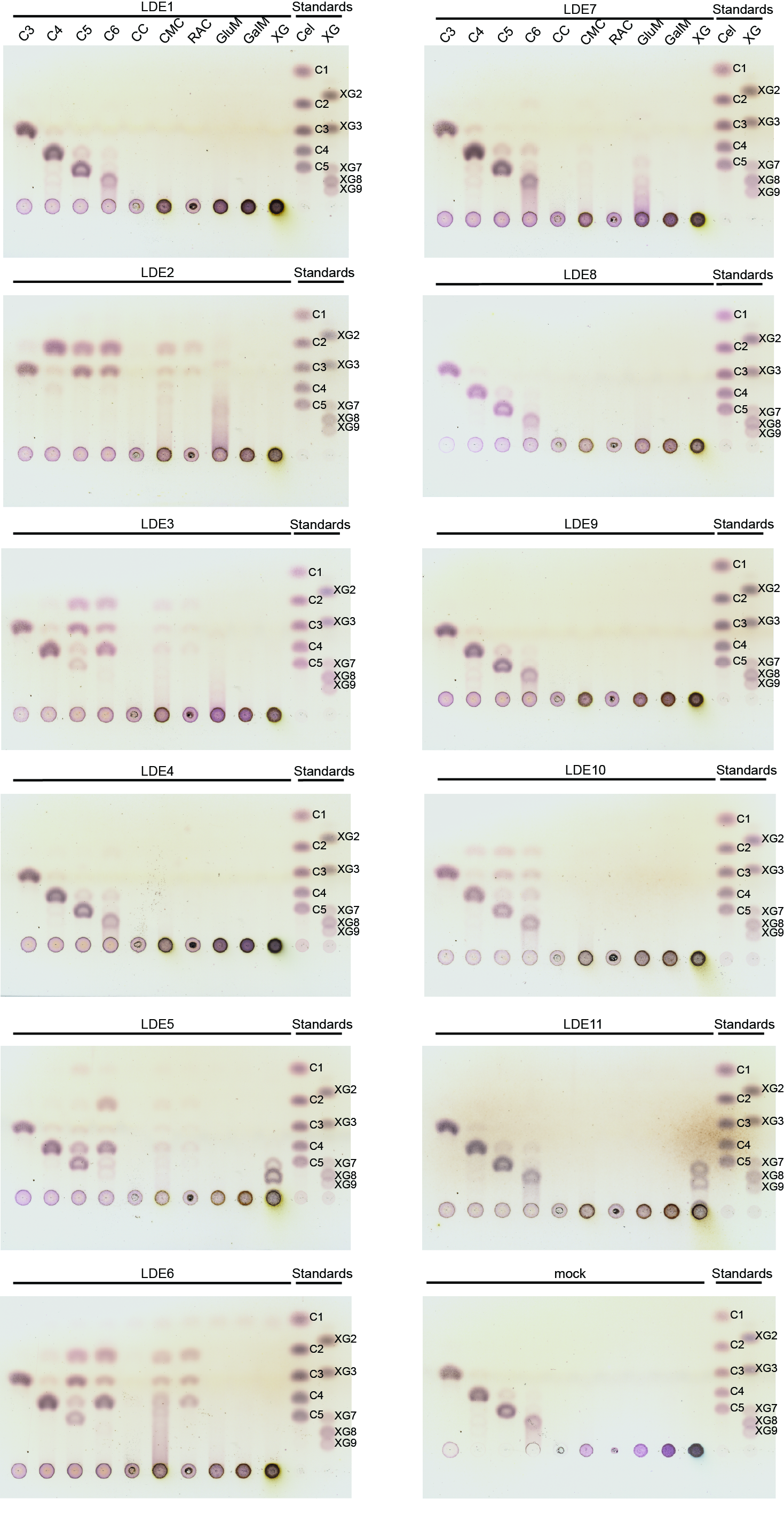


**Fig. S3 continued**

**
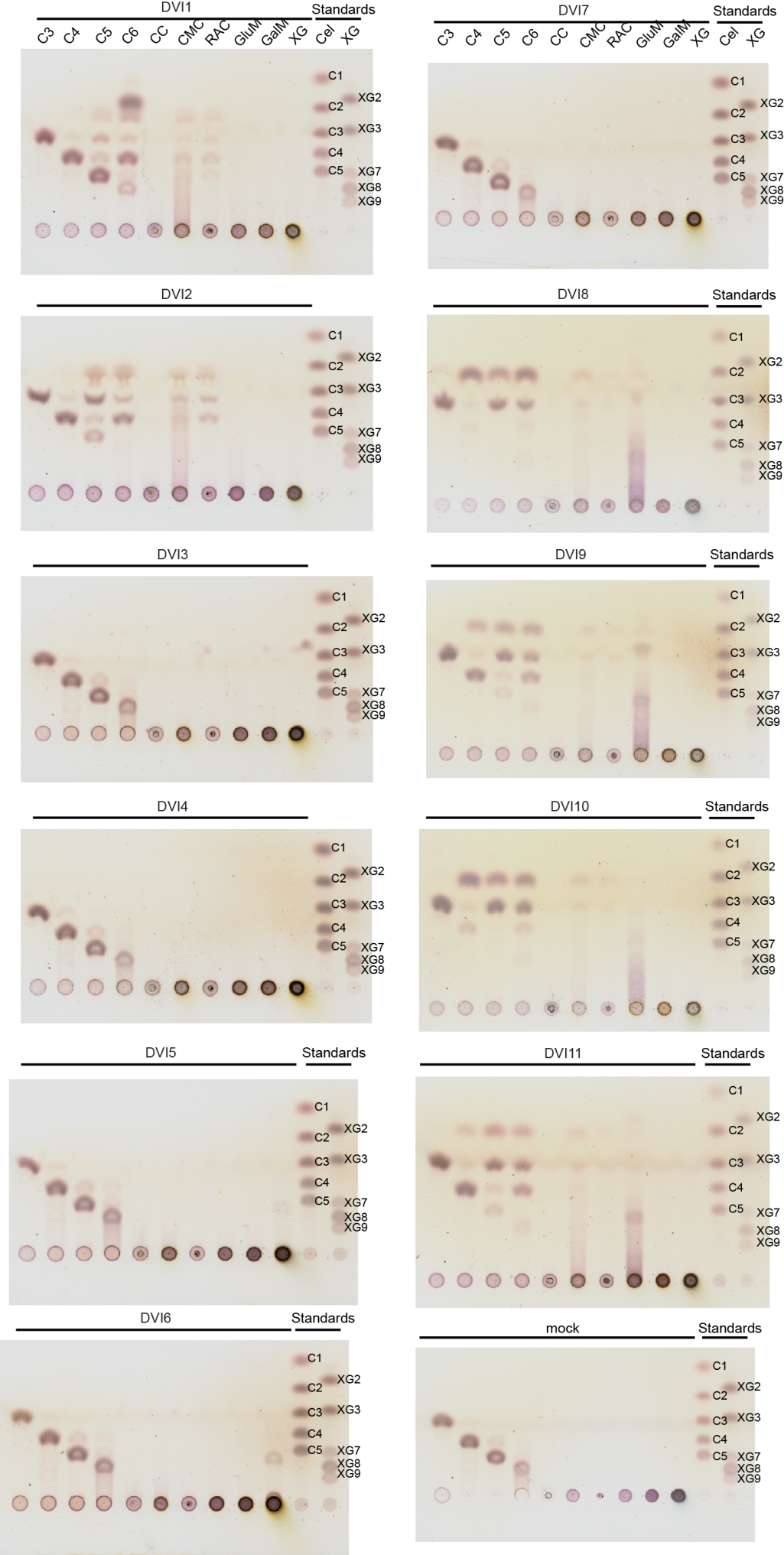
**

**Fig. S4 Thin-layer chromatography of *D. virgifera* GH45s assayed against several plant cell wall polysaccharides.** Recombinant GH45s were incubated for 16 h at 40 °C with various plant polysaccharides. Their breakdown products were analyzed on TLC and visualized using 0.2 % orcinol in methane/sulphoric acid (9:1) under continuous heating. Each TLC represents an individually tested GH45 (Dvi1 to Dvi11). All GH45s were assayed against the same set of substrates: cellotriose to cellohexaose (C3-C6); crystalline cellulose = avicel (CC); carboxymethyl cellulose (CMC); regenerated amorphous cellulose (RAC); glucomannan (GluM); galactomannan (GalM); xyloglucan (XG); standards: C1 = Glucose, C2 – C5 = cellobiose – pentaose; XG2- XG9 = xyloglucan- oligomers.


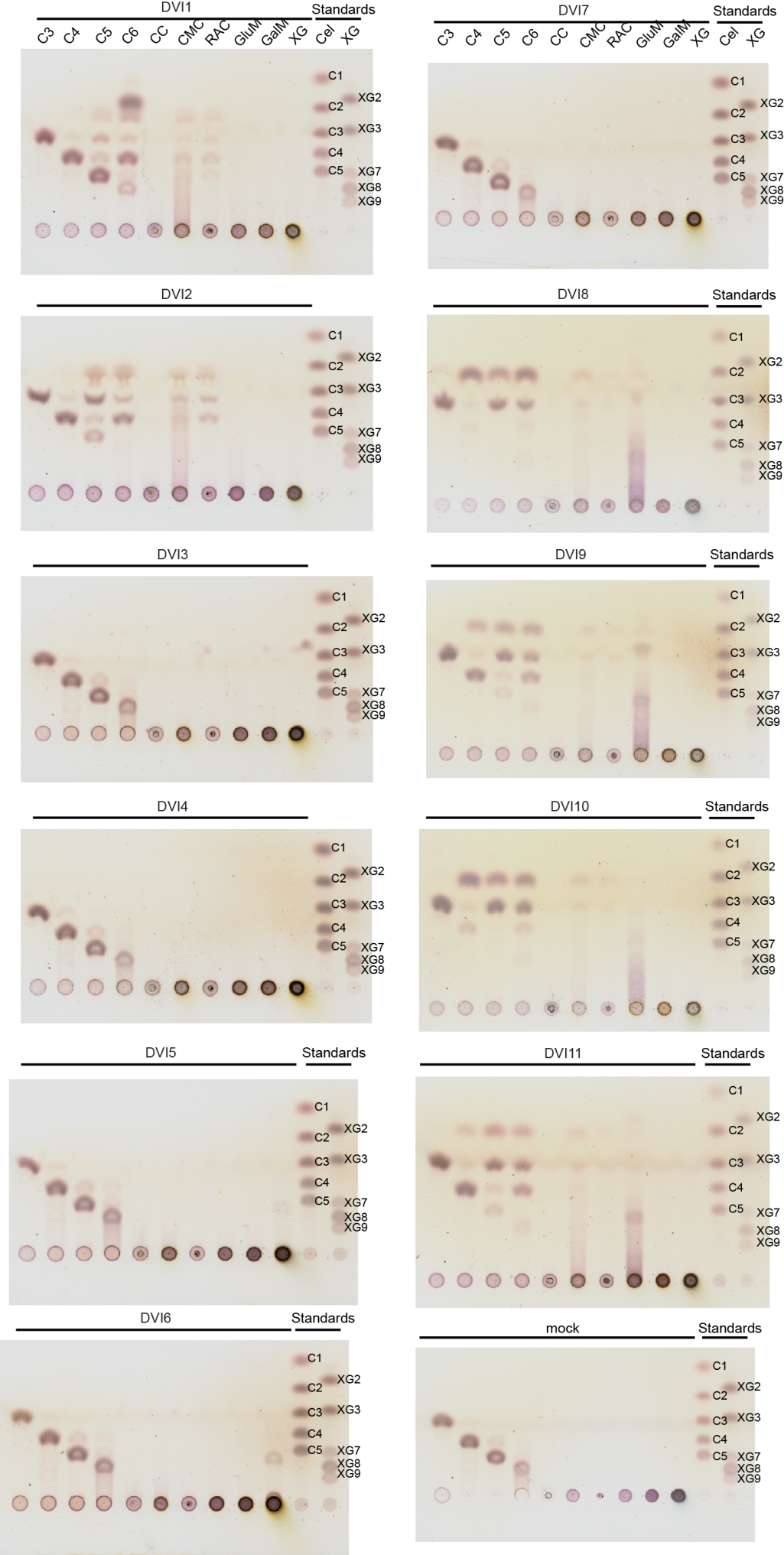


**Fig. S4 continued**

**
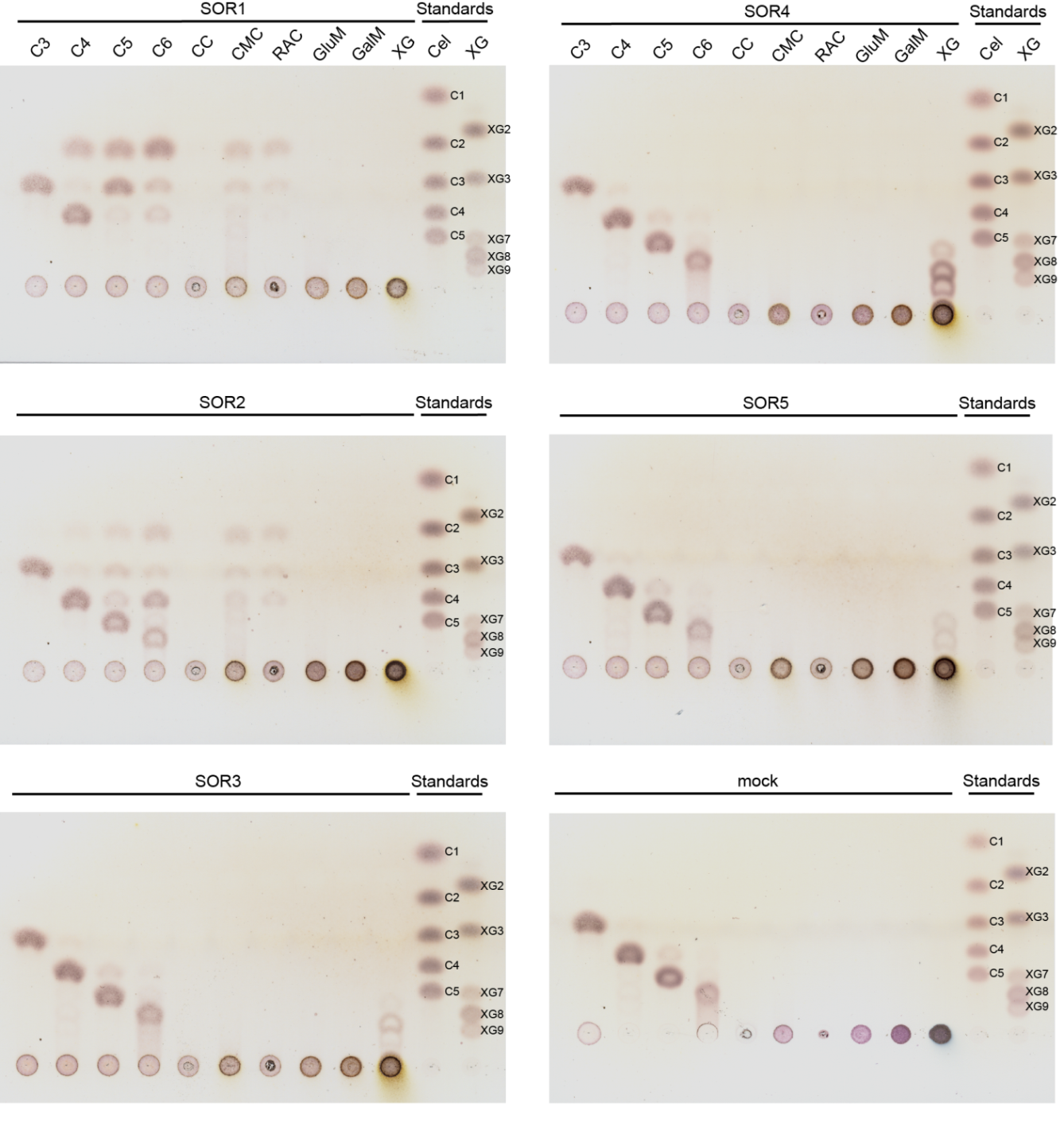
**

**Fig. S5 Thin-layer chromatography of *S. oryzae* GH45s assayed against several plant cell wall polysaccharides.** Recombinant GH45s were incubated for 16 h at 40 °C with various plant polysaccharides. Their breakdown products were analyzed on TLC and visualized using 0.2 % orcinol in methane/sulphoric acid (9:1) under continuous heating. Each TLC represents an individually tested GH45 (Sor1 toSor5). All GH45s were assayed against the same set of substrates: cellotriose to cellohexaose (C3-C6); crystalline cellulose = avicel (CC); carboxymethyl cellulose (CMC); regenerated amorphous cellulose (RAC); glucomannan (GluM); galactomannan (GalM); xyloglucan (XG); standards: C1 = glucose, C2 – C5 = cellobiose – pentaose; XG2- XG9 = xyloglucan-oligomers.

**Fig. S6 Bayesian global phylogenetic analysis encompassing GH45 proteins from various taxa (expanded version of Fig. 4).** 264 GH45 sequences of microbial and metazoan origin were initially collected (see Methods) and their redundancy was eliminated at 90 % sequence similarity, resulting in a total of 201 sequences. Sequence details are given in Table S2. Fungal branches are marked in orange, symbiotic protists in red, Collembola, Oribatida and Entognatha in dark blue, Coleoptera in light blue, Nematoda and Tardigrada in dark green, Rotifera in light green and bacteria in purple.

**Fig. S7 Maximum likelihood inferred phylogenetic analysis encompassing GH45 proteins from various taxa.** 264 GH45 sequences of microbial and metazoan origin were initially collected (see Methods), and their redundancy was eliminated at 90 % sequence similarity, resulting in a total of 201 sequences. Sequence details are given in Table S2. Fungal branches are marked in orange, symbiotic protists in red, Oribatida in dark blue, Collembola, Entognatha and Coleoptera in light blue, Nematoda in light green, Tardigrada and Rotifera in dark green and bacteria in purple.


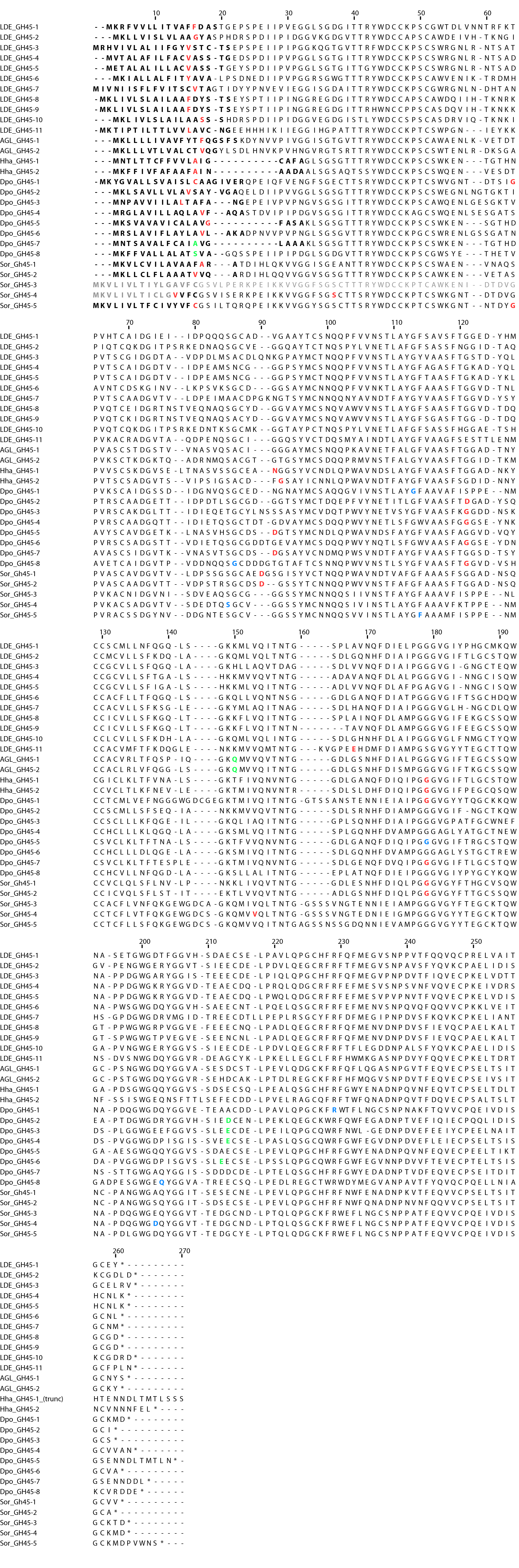


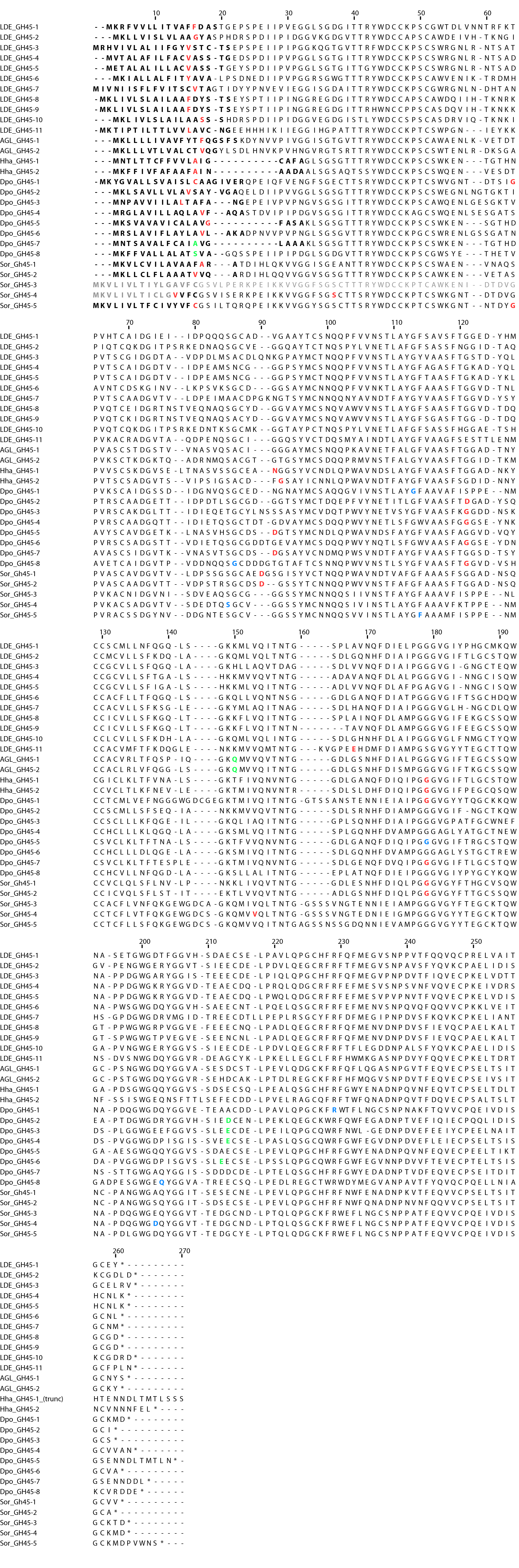


**Fig. S8 Conservation of intron position in phytophagous beetles with known GH45 genome structure.** Amino acid alignment of GH45 sequences derived from four different phytophagous beetles using MUSCLE. Genomic sequence information was retrieved from the genome assemblies of *L. decemlineata* (Schoville et al., 2018), *H. hampei* (Vega et al., 2015), *A. glabripennis* (McKenna et al., 2016) and *D. ponderosae* (Keeling et al., 2013). The predicted signal peptide is marked in bold letters. Intron positions are highlighted by colored amino acids according to their phase. Phase 0: green; Phase 1: red; Phase 2: blue.


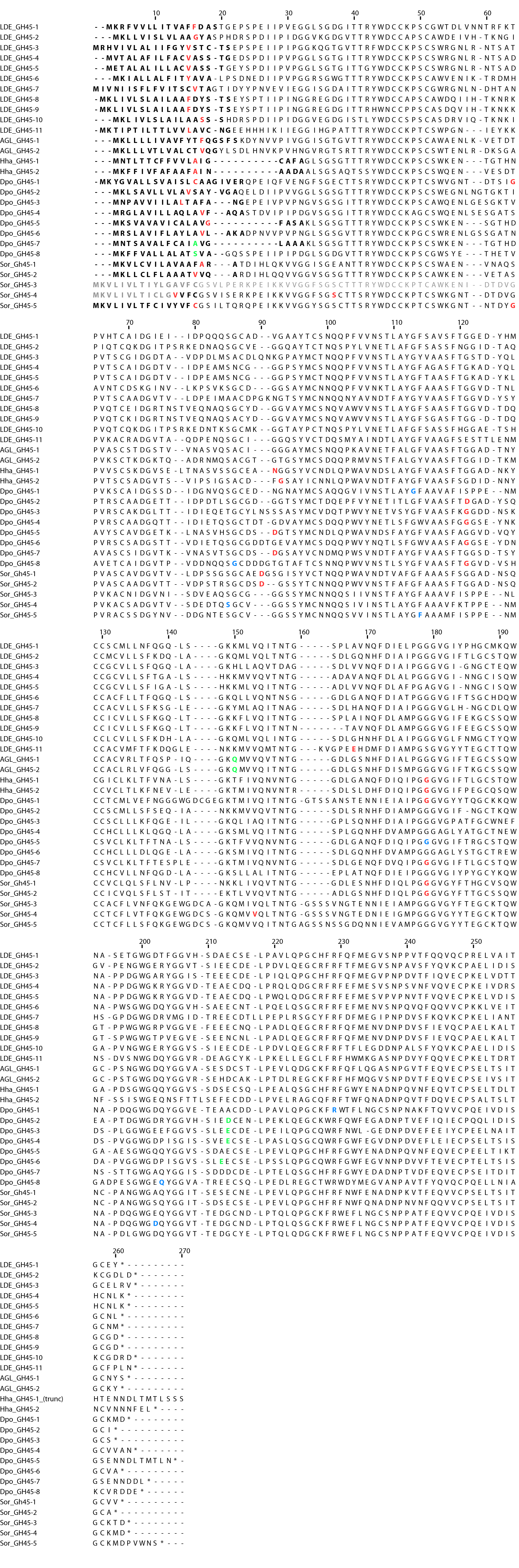
**Fig. S8 continued**

**
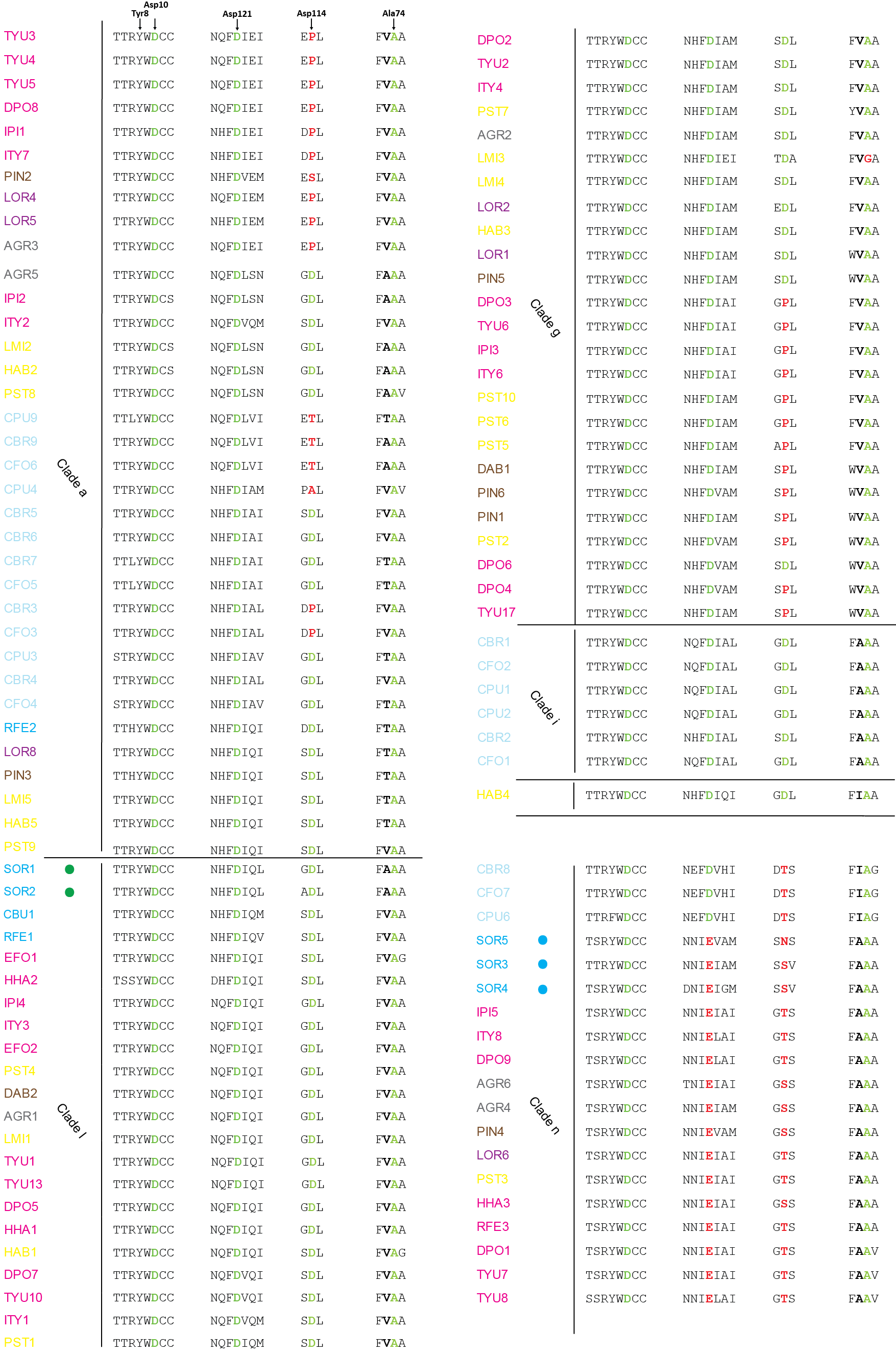
**

**Fig. S9 Amino acid alignment of the GH45 catalytic residues based on our Curculionoidea-based phylogeny.** We used a GH45 sequence of Humicola insulens (HIN1) as a reference sequence (Accession: 2ENG_A) (Davies, et al. 1995). According to HIN1, we chose to investigate the catalytic residues (ASP10 and ASP121) as well as a conserved tyrosine (TYR8) of the catalytic binding site, a crucial substrate stabilizing amino acid (ASP114) and an essential conserved alanine (ALA74). Arrows indicate amino acid residue under investigation. If highlighted in green, the residue remained unchanged in comparison to HIN1; elsewise it is highlighted in red. GH45 enzymatic activity was color-coded based on the respective substrate specificity (green dots = endo-β-1,4-glucanase, blue dots = endo-β-1,4-xyloglucanase, red dots= no activity). Color coding in reference to the respective subfamily: pink = Scolytinae (Curculionidae); brown = Entiminae (Curculionidae); purple = Cyclominae (Curculionidae); gray = Curculioninae (Curculionidae); yellow = Molytinae (Curculionidae); light blue = Brentinae (Brentidae); dark blue = Dryophthorinae (Curculionidae). Each clade corresponds to the clades depicted in Fig. 4.

**
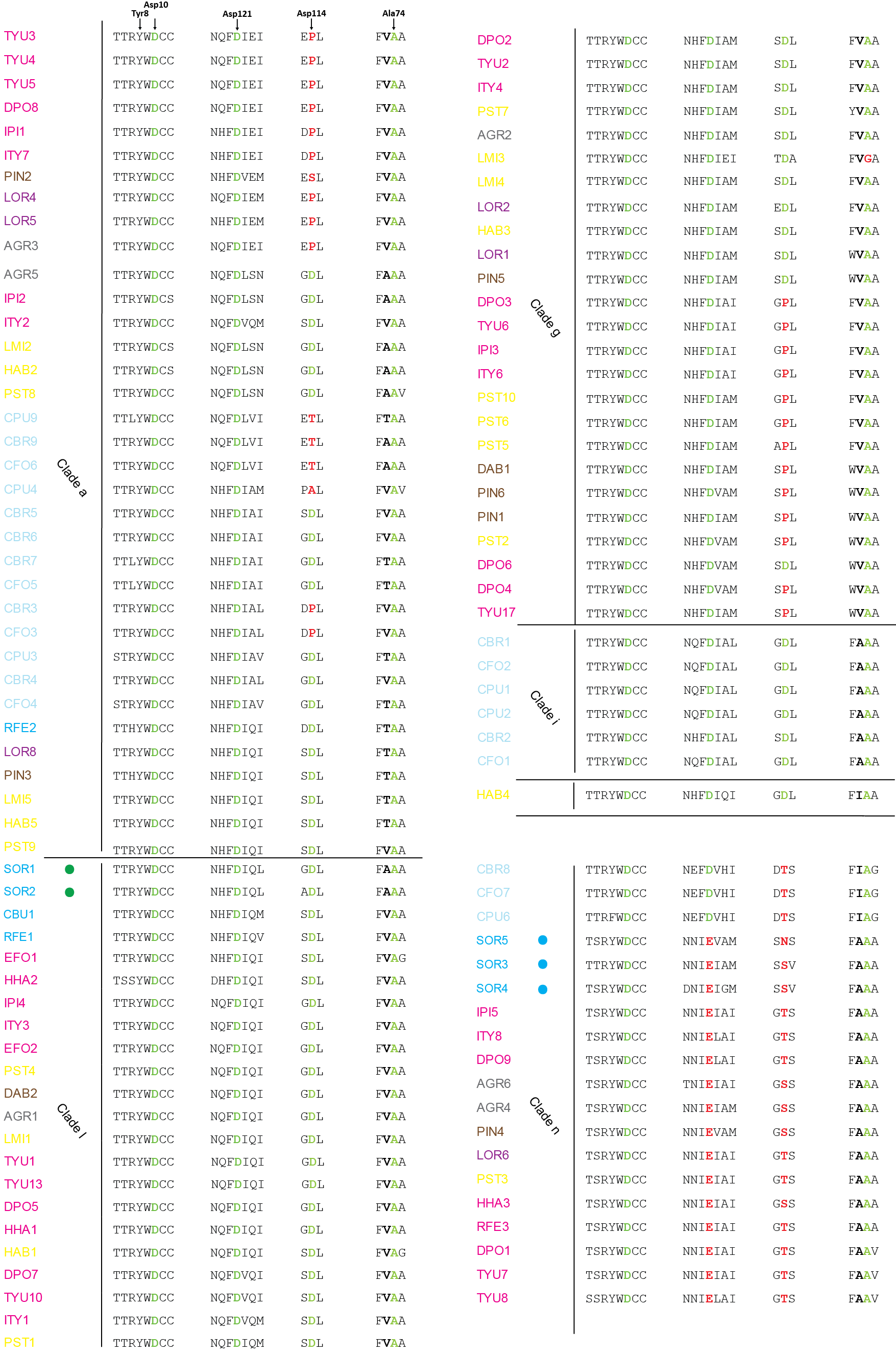
**

**Fig. S9 continued**


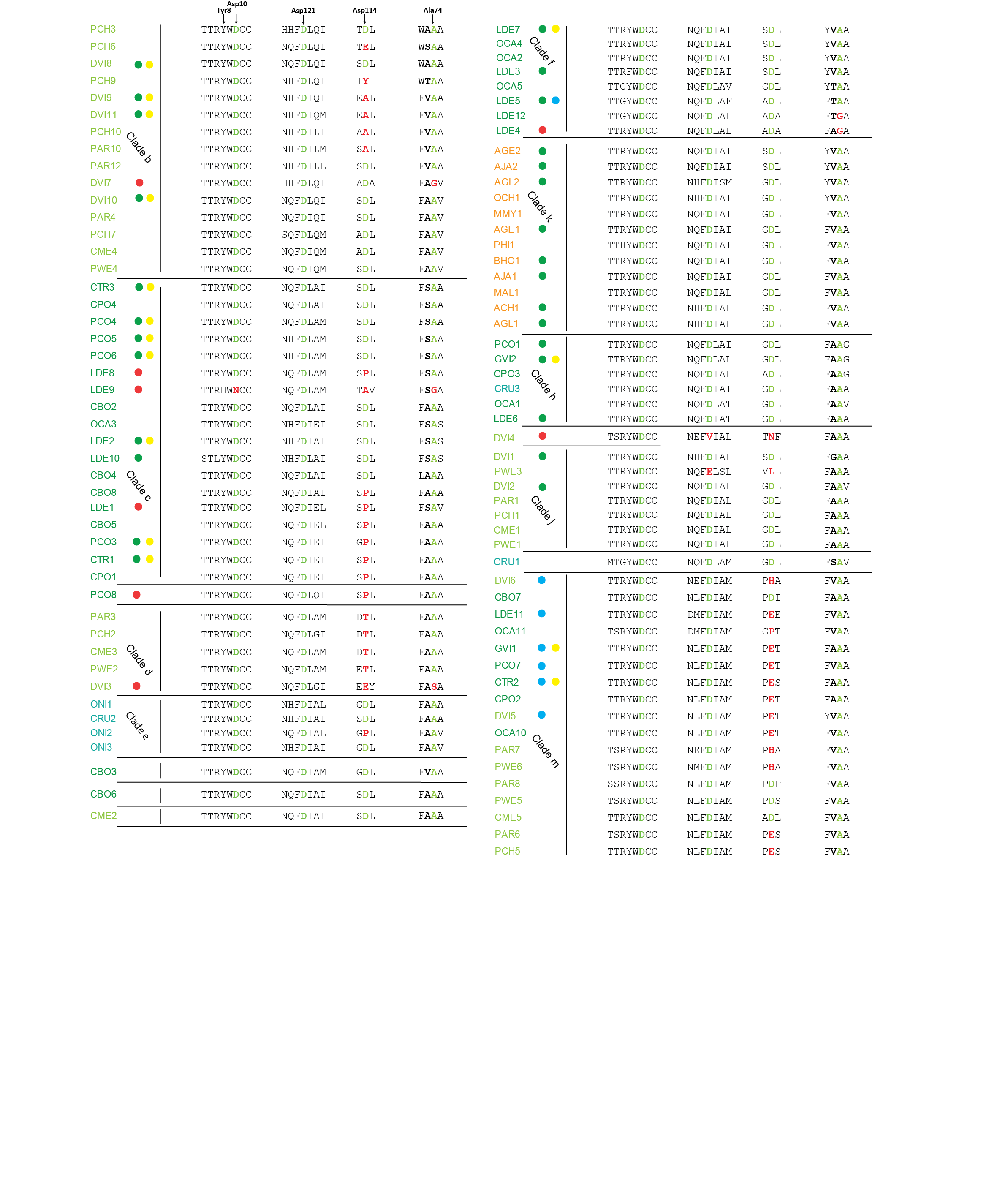


**Fig. S10 Amino acid alignment of the GH45 catalytic residues using our Chrysomeloidea-based phylogeny.** We used a GH45 sequence of Humicola insulens (HIN1) as a reference sequence (Accession: 2ENG_A) (Davies et al., 1995). According to HIN1, we chose to investigate the catalytic residues (ASP10 and ASP121) as well as a conserved tyrosine (TYR8) of the catalytic binding site, a crucial substrate stabilizing amino acid (ASP114) and an essential conserved alanine (ALA74). Arrows indicate amino acid residue under investigation. If highlighted in green, the residue remained unchanged in comparison to HIN1, otherwise it is highlighted in red. GH45 enzymatic activity was color-coded based on the respective substrate specificity (green dots = endo-β-1,4-glucanase, blue dots = endo-β-1,4-xyloglucanase, red dots = no activity). Color-coding was performed with reference to the respective subfamily: dark green = Chrysomelinae (Chrysomelidae); light green = Galerucinae (Chrysomelidae); orange = Lamiinae (Cerambycidae); cyan = Cassidinae (Chrysomelidae). Each clade corresponds to the clades depicted in Fig. 4.
